# Competition and Training Strategies for Developing World Class 200- and 400-m Individual Medley Swimmers

**DOI:** 10.5114/jhk/167381

**Published:** 2023-07-06

**Authors:** José María González-Ravé, José Antonio del Castillo, Jesús Santos del Cerro, Francisco Hermosilla, David B. Pyne

**Affiliations:** 1 Faculty of Sports Sciences, Sports Training Laboratory, University of Castilla La Mancha, Toledo, Spain.; 2 National Institute of Physical Education, Barcelona, Spain.; 3 Faculty of Law and Social Sciences, University of Castilla La Mancha, Toledo, Spain.; 4 Department of Physical Activity and Sports Science, Alfonso X El Sabio University, Madrid, Spain.; 5 Faculty of Life and Nature Sciences, Nebrija University, Madrid, Spain.; 6 Faculty of Health, Research Institute for Sport and Exercise, University of Canberra, Bruce, Australia.

**Keywords:** elite, swimming, strokes, middle distance

## Abstract

Swimming performance achieved in 50-m, 100-m and 200-m events in each swimming stroke can have an influence on the final performance in individual medley (IM) events. We attempted to quantify the relative contributions of performance in individual stroke events to top-10 world ranked IM competition performance. We examined competition results of top-10 world ranked IM swimmers (90 males and 90 females) between 2012 and 2018. A general linear model was developed to examine association between the 200-m and 400-mIM and predictor variables of competition performance in other 50-m, 100-m, and 200-m events. The main predictor variable for 200-mIM medalist status was having scored more than 900 FINA points in at least one 100-m event. Scoring more than 800 FINA points in at least two 200-mIM events, and more than 900 FINA points in at least one 100-m event, was important for success in the 400-mIM. Top-10 world ranked 200-mIM and 400-mIM swimmers require a world class standard in one or more individual stroke event(s)

## Introduction

Despite an increasing amount of research devoted to middle-distance events in a variety of sports, information regarding the training methodologies and competition strategies of world-class swimmers is limited. There is a lack of studies on the race analysis, periodization of training, and preparation strategies in 200-mIM and 400-mIM events. In contrast, there is more information in 100 m and 200 m events in the four strokes of butterfly, backstroke, breaststroke, and freestyle (Gonjo and Olstad, 2021). Competing in different events for IM swimmers throughout the season is an important component of their preparation for international events (González-Ravé et al., 2022; Lipińska 2011). Typically coaches emphasize longer distance events in the early season, and then shift to shorter distance events closer to competition (McGibbon et al., 2020).

The international federation (FINA), national federations, as well as coaches and team personnel use a standardized scoring system to rank national teams and swimmer performances. The FINA Points system permits comparisons of results between genders, different events, and different individual swimmers. The FINA points score system rates each race performance based on the current world record ratified by FINA. A score of 900 FINA points is the threshold usually assigned to world-class performances, with fewer points for slower performances (Garrido et al., 2012). World class swimmers usually score up to 900 or more FINA points in their main event (Formosa et al., 2013). Elite or international-level swimmers score around 850–900 FINA points in their main event, whereas national swimmers achieve a performance standard under 800 points (Mallett et al., 2021). The base times are defined for all common individual events and relays, separated for men/women and long course/short course pools.

König et al. (2014) define world class level swimmers as finalists of international events such as the FINA World Championships and the Olympic Games. In this case, we narrowed the definition of successful swimmers to top-10 ranked swimmers who are in contention for an Olympic medal (Trewin et al., 2004). We now seek to extend these selected reports to a comprehensive and detailed analysis of IM swimming at an international level over an extended period.

The 200-mIM and 400-mIM are the most challenging events in swimming, and the complexity in their preparation gives them a special appeal (Del Castillo et al., 2022; Hermosilla et al., 2021). Analysis of the last decade of international swimming shows the breadth and depth of various world-ranked IM swimmer profiles. Depending on the IM event (200 and 400 m), swimmers should achieve 700–900 FINA points in 100 and 200 m events of each stroke (Del Castillo et al., 2022).

To achieve the best performance in the 400-mIM, coaches must ensure that middle-distance front crawl training is a priority given a positive association between freestyle and IM swimming (Del Castillo et al., 2022). To improve IM swimmers’ performance, it is important to understand factors contributing to competition performance in both the 200-mIM and 400-mIM events for effective training planning, prescription, and monitoring. The number of other events of each stroke (not only IM) in which a swimmer will compete through the season should be a priority for coaches (Hermosilla et al., 2021). However, detailed guidelines are lacking on whether to prioritise the different freestyle and form stroke events, and choice of distances for interval training prescription.

The distribution of events in which an IM swimmer can score FINA points commensurate with world-class standard would help understand the profile of the best 200-mIM and 400-mIM swimmers. Therefore, the aim of this study was to develop a profile of the top-10 world ranked IM swimmers for both males and females, over a representative period of the Olympic Games, World Championships, and international competitions, according to the FINA points in all events in which they participated.

## Methods

### Participants

Our own customized database was developed using historical data from websites containing official results. First, the top-10 swimmers of the FINA World Ranking (Long Course) were selected from the website http://www.fina.org/ (accessed on 10 January 2022) for 200-mIM and 400-mIM (male and female) over 7 years from 2012 to 2018 inclusive. This period included the 2012 London and 2016 Rio Olympic Games, and the 2013 (Barcelona), 2015 (Kazan) and 2017 (Budapest) FINA World Championships. Secondly, after selecting the swimmers, we searched the website http://www.swimrankings.net (accessed on 10 January 2022) for the competitive performances (time) of each swimmer in the rankings for all individual events, including competitive events of 50, 100, 200 m for any stroke that the swimmer completed.

### Measures

The following variables were analysed: n50, number of 50 m events scoring more than 800 FINA points; n100, number of 100 m events scoring more than 800 FINA points; n200, number of 200 m events scoring more than 800 FINA points; Over100_900, a dichotomous variable assigned the value of 0 or 1 depending on whether for a given 100 m performance the score was higher than 900 FINA points or not; and Over200_900, also a dichotomous variable with a value of 0 or 1 depending on whether for a given 200-m competition performance the score was higher than 900 FINA points or not.

The final data comprised 140 and 140 entries in the 200-mIM and 400-mIM, respectively, from 90 international swimmers (male and female). The Nebrija University Ethics Committee approved this research project (application number FGM02102019), and since the data were based solely on publicly available resources, no informed consent was sought. All methods were performed in accordance with the relevant guidelines and regulations.

### Statistical Analysis

A general linear model (GLM) was conducted to examine the links between the dependent variables 200-mIM and 400-mIM, and the predictor variables. For each group and gender, specific models were developed, and the R^2^ coefficient calculated. The analysis was developed to determine whether variables included in the models influenced the FINA points in the 200-mIM and 400-mIM events. The β coefficient was calculated to determine the degree of change in the independent variable when one dependent variable was modified. A *p*-value <0.05 in the linear regression model was considered significant. Descriptive statistics were used to analyse the explanatory variables.

All the residuals showed a satisfactory distribution pattern. In addition, a non-parametric classification method, decision tree analysis, was employed to provide a graphic representation of finalists and medalists. In this analysis, the total sample was divided into two sub-samples, a learning sample to estimate both models, and a validation or test sample for subsequent validation of the models. Statistical analyses were conducted using R software (version 4.1.2 for Windows).

## Results

The influence of the 50-m, 100-m, and 200-m events with FINA points equal or higher than 800 on 200-mIM performance based on the explanatory variables is presented in Table 1. The R^2^ values were 0.36, 0.34 and 0.41, respectively, for all IM swimmers, both females and males, indicating an acceptable degree of goodness of fit, and therefore a model with good explanatory power.

**Table 1:** General linear model for determining the contribution of 50-, 100-, and 200-m events to top-10 world ranked performances in the 200-mIM. A positive β value indicates an improvement in performance, whereas a negative value indicates a reduction in performance.

GLOBAL					
	Estimate (β)	Std.Error	t value	*p*-value	R^2^
**(Intercept)**	896	12	74	<2e-16 *†*	0.36
**Age**	0.52	0.5	0.99	0.32	
**N50**	4.9	3.1	1.59	0.11	
**N100**	7.5	2.0	3.79	0.0002 *†*	
**N200**	3.6	1.8	2.01	0.047*	
**Over900_100**	14.5	5.6	2.60	0.015*	
**Over900_200**	6.6	4.1	1.63	0.11	
**FEMALES**					
**(Intercept)**	909	19	48	<2e-16	0.34
**Age**	0.16	0.9	0.19	0.85	
**n50**	3.4	4.8	0.71	0.48	
**n100**	8.8	3.2	2.8	0.01	
**n200**	3.8	2.5	1.5	0.14	
**Over900_100**	16	8.4	2.0	0.05	
**Over900_102**	2.1	6.4	0.33	0.74	
**MALES**					
**(Intercept)**	878	18	46	<2e-16 *†*	0.41
**Age**	1.3	0.8	1.7	0.09	
**n50**	−7.5	5.4	−1.4	0.17	
**n100**	5.0	2.7	1.8	0.07	
**n200**	4.9	2.8	1.7	0.09	
**Over900_100**	12	7.8	1.7	0.10	
**Over900_200**	9.6	5.6	1.7	0.09	

*Note:* R2: proportion of variance explained for 200-mIM competition performance; Age: age in years of each swimmer; n50: number of 50-m events scoring more than 800 FINA points; n100: number of 100-m events scoring more than 800 FINA points; n200: number of 200-m events scoring more than 800 FINA points; Over100_900: dichotomous variable with a value of 0 or 1. It depends on whether in any of the 100-m tests the score was higher than 900 FINA points or not; Over200_900: dichotomous variable with a value of 0 or 1. Significance. codes: †: p = 0.000; *: p < 0.05

There was a strong relationship between 100-m events in 200-mIM (β = 7.5, *p* = 0.00), as well as in 200-m events (β = 3.6, *p* = 0.04), and the final performance, which influenced the position of the top-10 FINA in the 200-mIM. Each additional 100-m event with FINA points equal or higher than 800 for a swimmer was associated with an increase of 7.5 FINA points in 200-mIM. Comparable results were evident for female swimmers (β = 8.8, *p* = 0.00). Moreover, a 100-m event with more than 900 FINA points was associated with an increase of 14.5 FINA points in the 200-mIM (*p* = 0.015).

Table 2 shows the influence of 50-m, 100- m, and 200-m with FINA points equal or higher than 800 on 400-mIM competition performance. Similar to the results in 200-mIM, the R^2^ values were 0.43, 0.49 and 0.42, respectively, again evidence of a moderate degree of goodness of fit, and a model with good explanatory power. Each additional year of age in a swimmer was associated with a reduction of 1 FINA point in the 400-mIM (β = −1.1 *p* = 0.03). Each additional 200-m event (freestyle, butterfly, backstroke, or breaststroke) with FINA points equal or higher than 800 was associated with an increase of 7 FINA points in the 400-mIM (*p* = 0.00). A 200-m event with more than 900 FINA points was associated with an increase of 13 FINA points in the 400-mIM (*p* = 0.00).

**Table 2:** General linear model for determining the contribution of 50-, 100-, and 200-m events to top-10 world ranked performances in the 400-mIM. A positive β value indicates an improvement in performance, whereas a negative value indicates a reduction in performance.

GLOBAL					
	Estimate (β)	Std.Error	t value	*p*-value	R^2^
**(Intercept)**	919	13	66.3	<2e-16 *†*	0.43
**Age**	−1.0	0.5	−2.1	0.03	
n50	9.1	4.9	1.9	0.07	
n100	3.9	2.2	1.8	0.08	
n200	7.0	1.5	4.7	7,59e-06 *†*	
Over900_100	−4.9	8.9	−0.6	0.58	
Over900_200	13.4	3.6	3.7	0.0003 *†*	
**FEMALES**					
**(Intercept)**	926	28	33	<2e-16 *†*	0.49
**Age**	−1.7	0.7	−2.4	0.02	
n50	7.8	14.4	0.6	0.54	
n100	4.0	3.9	1.0	0.32	
n200	5.9	2.2	2.7	0.01 *†*	
Over900_100	8.5	24.2	0.3	0.73	
Over900_200	9.8	5.1	1.9	0.06	
**MALES**					
**(Intercept)**	905	19	47	<2e-16	0.43
**Age**	−0.30	0.7	−0.5	0.61	
n50	5.8	7.6	0.8	0.45	
n100	3.1	2.8	1.1	0.27	
n200	8.2	2.2	3.7	00005 *†*	
Over900_100	−10.9	10.3	−1.1	0.29	
Over900_200	17.7	5.3	3.3	00014 *†*	

*Note:* R2: proportion of variance explained for 400-mIM competition performance; Age: age in years of each swimmer; n50: number of 50-m events scoring more than 800 FINA points; n100: number of 100-m events scoring more than 800 FINA points; n200: number of 200-m events scoring more than 800 FINA points; Over100_900: dichotomous variable with a value of 0 or 1. It depends on whether in any of the 100-m tests the score was higher than 900 FINA points or not; Over200_900: dichotomous variable with a value of 0 or 1. *Significance. codes: †: p = 0.000*

Finally, we analysed the pattern of FINA points in different strokes of 100-m events for 200-mIM, and similarly, the FINA points in 200-m events for the 400-mIM. Table 3 shows the mean FINA points for finalists and medalists in the different swimmers’ profiles for both IM events. Swimmers needed ~20–60 higher FINA points in 100-m events to be confident of medal performance in the 200-m IM, and ~10–40 higher FINA points in 200-m events to improve their chance of a medal in the 400-m IM.

**Table 3:** Mean FINA points in 100-m events and 200-m events for 200- and 400-mIM swimmers and difference between a finalist and a medalist.

		100-m events			
		Butterfly	Backstroke	Breaststroke	Freestyle
200-mIM	Mean FINA points finalist	799	834	801	833
	Mean FINA points medalist	866	889	794	852
	Difference in FINA points between finalist and medalist	66	55	−7	19
		**200-m events**			
		Butterfly	Backstroke	Breaststroke	Freestyle
400-mIM	Mean FINA points finalist	833	813	823	840
	Mean FINA points medalist	875	857	846	851
	Difference in FINA points between finalist and medalist	42	44	23	12

**Figure 1:**
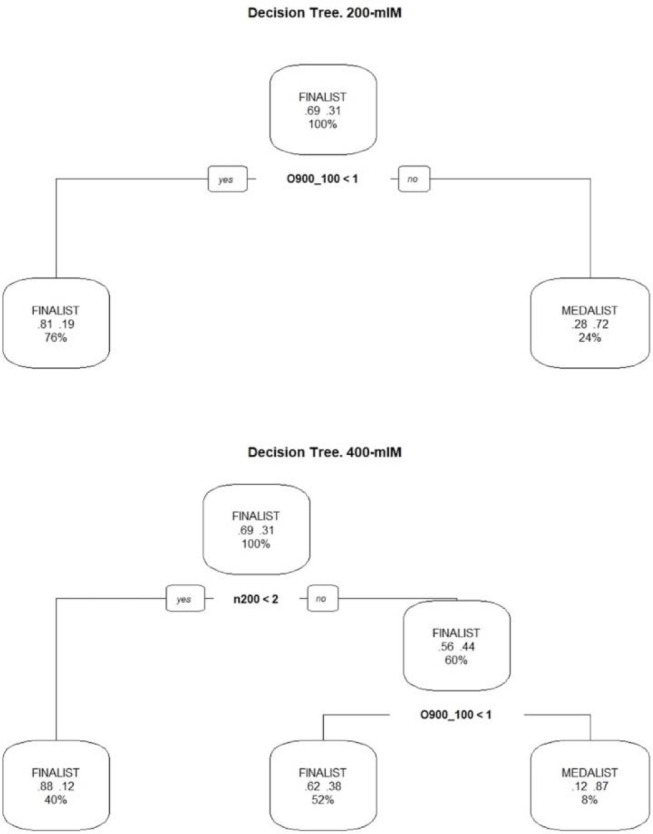
Decision tree analysis showing the proportion of finalists and medalists relative to a threshold value of 900 FINA points in the 200mIM (upper tree reflecting 100-m events) and 400mIM (lower tree reflecting performance in 200-m events).

The decision trees illustrated the following results for IM swimmers in a medal position (Figure 1). For the 200-mIM events, the main predictor variable for medalist status was having scored more than 900 FINA points in at least one 100-m event. From the model validation sample, the accuracy coefficient for this tree was 80%, which is an acceptably good result. In the 400-mIM, to be a medalist, it was necessary to have scored more than 800 FINA points in at least two 200-m events, and more than 900 FINA points in at least one 100-m event. For this tree, the accuracy coefficient from the validation sample was 74%.

## Discussion

The importance of the ability to swim the individual events (freestyle, butterfly, backstroke, and breaststroke) to a high standard is acknowledged by all IM coaches and swimmers. We have shown here that ~40% of the variation in international 200-m and 400-m IM competition performance by top-10 World Ranked swimmers can be attributed to recent performance in individual 50-m, 100-m, and 200-m events. The main predictor variable for medalist status was having scored more than 900 FINA points in at least one 100-m event. This information is useful for coaches so that they can adopt a seasonal strategy of participating in different 100-m and 200-m events, in different form strokes, prior to the main competition of the season. Coaches and swimmers should consider the value of training and competing in a range of events across strokes (freestyle, form stroke, medley) and distances (50 m to 200 m) to improve subsequent IM performance.

These results confirm the assertions of Mujika et al. (2019) who indicated that swimmers should be encouraged to elevate their world ranking in a competitive season, then concentrate on improving that performance to have a realistic chance of a medal at a major international competition. The analysis of Trewin et al. (2004) supports the notion that swimmers are more consistent between distances with the same stroke, than between strokes of the same distance. Each additional 100-m event with FINA points equal or higher than 800 in a swimmer was associated with an increase of 7 FINA points in 200-mIM, and a 100-m event with more than 900 FINA points yielded an increase of 15 FINA points in the 200-mIM. Comparable results were obtained for the 400-mIM. Each additional 200-m event with FINA points equal or higher than 800 achieved by a swimmer was associated with an increase of 7 FINA points in 400-mIM. Each 200-m event with more than 900 FINA points was associated with an increase of ~13 FINA points in the 400-mIM. Coaches should develop, refine, and evaluate a within- and between-season training plan to develop competition performance in both the 200- and 400-m IM events, as shown in a study of Nugent et al. (2017).

In general, 200-mIM specialists hold higher FINA points in sprint swimming, while 400-mIM specialists hold higher FINA points in middle distance events (Del Castillo et al., 2022). González-Ravé et al. (2022) presented a case study of periodized training of a world-class 400-m Individual Medley (IM) swimmer (4^th^ in the 2019 World Championships) in the season culminating in a bronze medal in the 2018 European Championship. This athlete had FINA points between 850 and 900 points in the 200-m butterfly and 400-mIM. The current analyses with 180 top-10 world ranked swimmers confirm the importance of strategically developing 50-, 100-, and 200-m performances. Swimmers can race alternate events early in the season to get race practice, then closer to the major competition event, selection becomes more specific to the swimmers’ priority events. This sequence follows the same pattern as the evolution of the training prescription throughout the season, where the specificity gradually increases (McGibbon et al., 2020). The best performance in a season is usually achieved by swimmers at the end of the season underpinned by expert prescription of training volume and intensity. Manipulation of training loads, volume and intensity is necessary for stimulation of adaptations through the overcompensation process (González-Ravé et al., 2021, 2022; Hellard et al., 2019; Stewart and Hopkins, 2000).

The pattern of FINA points in different strokes of 100-m and 200-m events for 200-mIM and 400-mIM has been also analyzed. For 200-mIM, to achieve a medal typically requires 834 FINA points in the 100-m backstroke, although the higher differences between a medalist and a finalist are in the 100-m butterfly (66 FINA points), highlighting the importance of that stroke, the lead-off stroke, in the IM events. For a 400-mIM swimmer, to achieve a medal requires 875 FINA points in the 200-m butterfly, also the largest difference (42 FINA points) between a medallist and a finalist in the same event. Our results confirm that the most demanding event in FINA points for the 200-mIM is the 100-m backstroke (Table 3). These results concur with the work of Saavedra et al. (2012) that suggests that the backstroke is the most determinant style for the final performance (of medalists) in both the 200-mIM and 400-mIM for men. In contrast, for the 400-mIM, the most demanding event is the 200-m freestyle (840 FINA points) and the 200-m butterfly (833 FINA points), respectively. This pattern of associations aligns with the results of Saavedra et al. (2012) for women, showing the same stroke (backstroke) in the 200-mIM, but freestyle in the 400-mIM.

## Conclusions

An elite 200-mIM swimmer needs to achieve more than 900 FINA points in one 100-m event to increase the likelihood of a medal at an international competition. Similarly, for the aspiring 400-mIM swimmer to be a medalist, it is necessary to have scored more than 800 FINA points in at least two 200 m events, and more than 900 FINA points in at least one 100-m event. The specificity and complexity of the IM requires both a well-planned and executed training program, and a carefully constructed schedule of events in minor and major competitions. The training program should comprise different 50-m, 100-m and 200-m intervals in a variety of strokes to support world-class IM swimming performance.
